# Comprehensive analysis of codon usage pattern in *Withania somnifera* and its associated pathogens: *Meloidogyne incognita* and *Alternaria alternata*

**DOI:** 10.1007/s10709-022-00154-w

**Published:** 2022-04-13

**Authors:** Jyoti Chandan, Suruchi Gupta, Vikash Babu, Deepika Singh, Ravail Singh

**Affiliations:** 1 CSIR-Indian Institute of Integrative Medicine, Jammu, 18000 India; 2 Academy of Scientific and Innovative Research (AcSIR), Jammu, 18000 India; 3 DZMB Senckenberg Am Meer, Wilhelmshaven, Germany

**Keywords:** Codon usage bias, Nematoda, Fungus, Plant, Parasite, Host

## Abstract

*Meloidogyne incognita* (Root-knot nematode) and *Alternaria alternata* (fungus) were among the dominant parasites of the medicinal plant *Withania somnifera*. Despite the fatal nature of their infection, a comprehensive study to explore their evolution and adaptation is lacking. The present study elucidates evolutionary and codon usage bias analysis of *W. somnifera* (host plant), *M. incognita* (root-knot nematode) and *A. alternata* (fungal parasite). The results of the present study revealed a weak codon usage bias prevalent in all the three organisms. Based on the nucleotide analysis, genome of *W. somnifera* and *M. incognita* was found to be A-T biased while *A. alternata* had GC biased genome. We found high similarity of CUB pattern between host and its nematode pathogen as compared to the fungal pathogen. Inclusively, both the evolutionary forces influenced the CUB in host and its associated pathogens. However, neutrality plot indicated the pervasiveness of natural selection on CUB of the host and its pathogens. Correspondence analysis revealed the dominant effect of mutation on CUB of *W. somnifera* and *M. incognita* while natural selection was the main force affecting CUB of *A. alternata*. Taken together the present study would provide some prolific insight into the role of codon usage bias in the adaptability of pathogens to the host’s environment for establishing parasitic relationship.

**Supplementary Information:**

The online version contains supplementary material available at 10.1007/s10709-022-00154-w.

## Introduction

*Withania somnifera* L. Dunal commonly known as Ashwagandha or Indian ginseng is a potent medicinal herb belonging to the family Solanaceae. It is well recognized as a rich resource of bioactive compounds that are used in more than 100 herbal formulations in Ayurveda, Siddha, and Unani systems of medicines (Lattoo et al. 2007). It possesses strong anti-inflammatory, antioxidant, immunomodulatory, anti-cancerous, anti-stress, and adaptogenic properties. The immense therapeutic potential of the plant is attributed to the presence of active constituents *i.e*. steroidal lactones (withanolides, withaferins) and acyl steryl glycosides (sitoindosides VII–X) in roots, leaves, and berries (Das et al. 2020). However, the annual production of *W. somnifera* roots is very less (2000 t) as compared to the annual requirement (7000 t), primarily due to the heavy phytopathogen infestation (Patra et al. 2004). Thus, the study of different factors, especially phytopathogens that limit the overall yield of a plant becomes very significant.

This plant is susceptible to several phytopathogens both under wild and cultivated conditions. Among them, the root-knot nematode, *Meloidogyne incognita* is a major constraint on the overall yield of *W. somnifera* leading to several visible and biochemical changes (Sikora and Schuster 2000; Saikia et al. 2013). Initial visible changes are indicated by galls and knots in roots that escalate into yellowing of leaves due to reduced uptake of nutrients such as P, K, Zn, Mn, and Cu on heavy infestation (Pandey et al. 2003; Saikia et al. 2013). Biochemical changes include alterations in the levels of amino acids, organic acids (Freire et al. 1985; Sikora and Schuster 2000) reduced chlorophyll (Ferraz et al. 1989) and carotenoid contents in plants. All this incurs huge economic loss by severely affecting plant’s dry weight parameters and secondary metabolite content (sominiferine, somnine, withanine, tropine, isopelletierine, cuscohygrine, anaferine, anahygrine, visamine, etc.) (Saikia et al. 2013). Also, nematode penetration causes wounding of roots which increases their susceptibility to subsequent fungal diseases (Taylor 1990).

In addition,  the plant is prone to infections by many fungal phytopathogens, such as *Alternaria alternata, Fusarium oxysporum, Myrothecium roridum,* etc. However, leaf spot disease caused by fungal pathogen *A. alternata* (Fr.) Keissler is the most prevalent disease leading to a drastic reduction in the bioactive content of plants (Pati et al. 2008). *A. alternata* infection causes various histological and biochemical changes in *W. somnifera* leaf. Both dorsal and ventral surfaces of leaf develop brown to black spots (2–9 mm) encircled by a yellow halo (Inoue and Hideo 2000; Mims et al. 2002).

Successful plant-fungus-plant-parasitic nematode (PPN) disease interaction depends on several factors like host genotype, nematode species, fungal population and environmental conditions (Back et al. 2002). Besides, codon usage bias (CUB) is an additional factor that influences host–pathogen interaction (Biswas et al. 2019; Deng et al. 2020; Gupta and Singh 2021). CUB is a phenomenon by which some codons are used more frequently than others during protein synthesis and gene expression (Di Paola et al. 2018). Mutational pressure, natural selection and random genetic drift are the three main factors that affect CUB (Tao and Yao 2020).

Synonymous substitution of codons affects the adaptive strategies and geographical distribution of the host by affecting mRNA splicing, transcription, protein synthesis and expression (Deb et al. 2021b, a). For successful invasion and association with the host, pathogens synthesize small secretive proteins in addition to degrading enzymes that help overcome the host defense mechanism (Badet et al. 2017). In this context, CUB largely determines favorable host–pathogen interaction by significantly affecting their translational efficiency (Sur et al. 2007; Sahoo et al. 2019; Arella et al. 2021). Codon optimization significantly affects the severity of pathogenic infection in a selective environment by influencing cellular growth that induces evolutionary changes in the pathogen for better adaptation to the host (Biswas et al. 2019).

Considering the huge economic loss incurred by these major pathogens on the overall yield of *W. somnifera* and lack of understanding on pathogenesis from a genomic perspective, the present study entails the comparative analysis of CUB pattern in host plant and selected pathogens with respect to its influence on host–pathogen interaction. The results of the current study would be helpful to build codon usage profiles of *W. somnifera* and selected pathogens for further genomic studies. Subsequently, it would lay a foundation for molecular genetic engineering studies to optimize pathogenesis-related genes either in *W. somnifera* or pathogens. Moreover, to the best of our knowledge, the genomic aspect of the above-mentioned interaction is investigated for the first time.

## Materials and methods

### Nematode and fungal sample isolation from *W. somnifera*

#### Nematode isolation

Nematodes were extracted from the three rhizosphere soil samples of *W. somnifera* plant by centrifugal floatation using MgSO_4_ at 1.18 specific density (Hooper et al. 2005). Nematodes were collected on a 20 mm sieve and thoroughly washed with sterile water. Isolated nematodes were fixed in hot 4% formalin, dehydrated in alcohol saturated chamber and processed to pure glycerine using Seinhorst’s method. Nematodes were transferred to a glass beaker and counted on a counting slide under an Olympus SZX12 stereomicroscope at 40–80 magnification. Permanent wax slides were prepared for light microscopy (LM) and morphometric studies of nematodes (Rizvi et al. 2010). Each specimen of nematodes was distinguished and identified based on morphological characters by following the literature.

#### Fungal isolation

Roots of *W. somnifera* were collected from the fields of CSIR-IIIM, Jammu. The harvested samples were brought to the laboratory in sterilized plastic bags and immediately processed for the isolation of fungal samples. The sample was washed and cleaned thoroughly under running tap water for 10–15 min to remove the dirt/debris. Further the sample was rinsed with different sterilization agents including 70% ethanol for 30 s, 1% sodium hypochlorite followed by rinsing 2–3 times with autoclaved distilled water. Finally sample was dried on filter paper and small explants were placed on plates containing potato dextrose agar (PDA) supplemented with antibiotic chloramphenicol (0.4 mg/100 ml). The Petri dishes were incubated at 26 ± 2 °C for 3 weeks and routinely monitored for any fungal growth (Salini et al. 2014). Pure cultures were obtained by transferring the hyphal tips emerging out of the plant tissue to fresh PDA plates. Pure cultures were maintained on PDA slants at 4ºC in the fungal germplasm collection at IIIM, Jammu. Among the isolated fungal samples from *W. somnifera*, the isolation frequency of RS-1 isolate was found to be maximum thus it was selected for further analysis.

### Identification of fungal isolate

Among all the isolates, identification of fungal isolate (RS-1) was carried out by studying their macroscopic and microscopic characteristics. The morphological features were compared by using relevant keys (St- Germain and Summerbel 1996; de Hoog et al. 2000). Further, for molecular characterization genomic DNA was isolated from the fungal strain by following the modified protocol of Saghai-Maroof et al. (1984). Mycelial mass was filtered through muslin cloth and dried to harvest the cells for DNA extraction. 500 mg of dried mycelial mass was ground in Pre-cooled mortar to a fine powder by using liquid nitrogen. Powdered mass was transferred to centrifuge cups containing 10 ml of pre- warmed (65 °C) CTAB extraction buffer (100 mM Tris HCl pH 8.0, 20 mM EDTA, 1.4 mM NaCl, 2% CTAB). Centrifuge tubes were incubated in water bath at 65 °C for 60 min. After incubation, equal volume of chloroform: isoamyl alcohol in the ratio of 24:1 was added followed by centrifugation at 7000 rpm for 10 min. Aqueous phase was transferred to another centrifugation tube to which double the volume of chilled ethanol was added. DNA was spooled out, washed with 70% ethanol, air dried and finally dissolved in TE. The universal primers ITS-1 and ITS-4 were used for the PCR amplification of 18S rDNA region. For this, 1 µl of purified and quantified DNA sample at a concentration of 50 ng/µl was used as a template in Thermal cycler. The purified PCR product was selected and sent for sequencing (Agrigenome labs Pvt. Ltd.). The generated ITS rDNA sequences were aligned, analysed and compared with Genbank database of NCBI (http://blast.ncbi.nlm.nih.gov/Blast.cgi, http://www.ncbi.nlm.nih.gov/genbank/) by BLAST search. Genbank accession number MW741555 was obtained by submitting confirmed sequence to Genbank (http://www.ncbi.nlm.nih.gov/genbank/submit).

### Retrieval of coding sequence data

All the protein-coding CDS (Coding Domain Sequences) sequences of host plant *W. somnifera* and its pathogens *M. incognita* (nematode) and *A. alternata* (fungus) were retrieved from National Centre for Biotechnology Information (NCBI) (https://www.ncbi.nlm.nih.gov/) in FASTA format. Out of all the complete CDS obtained from NCBI database using keywords “species name and complete CDS” 324, 261 and 234 coding sequences belonging to *W. somnifera*, *M. incognita*, and *A. alternata* sequentially with assigned gene names were selected for CUB analysis. However, CDS having length > 100 bp, devoid of perfect start and stop codons and having ambiguous codons were not considered for further analysis in order to avoid anomalies due to partial and short sequences and to minimize sampling errors. Also, the codons ATG (methionine), TGG (tryptophan), and three stop codons (UUA, UGA, UAG) were excluded for CUB analysis. To conclude, after removing undesired sequences, the total number of CDS employed in this study were 238, 160, and 207 for *W. somnifera, M. incognita* and *A. alternata* respectively. Further, the detailed information of the selected and excluded CDS is provided in Supplementary files S1 and S2 respectively.

### Nucleotide composition analysis

The coding sequences (CDS) of *W. somnifera* and its pathogens *M. incognita (*nematode) and *A. alternata* (fungus) were subjected to nucleotide composition analysis that included the frequencies of four nucleotide (A, T, G, C); frequency of nucleotides at third position of synonymous codons (A3, T3, G3, C3); % GC content at first (GC1), second (GC2) and third (GC3) codon positions and overall % GC content. Further, the percentage of GC12 value for each CDS was also calculated.

### Relative synonymous codon usage (RSCU) and preferred codon analysis

Relative synonymous codon usage **(**RSCU) is an important parameter to study the characteristics of CUB in an organism. It is equivalent to the ratio of observed frequency of codon to the expected frequency (Yang et al. 2014). Codon usage is considered as unbiased if RSCU = 1, positive if RSCU > 1 and negative if RSCU < 1. Similarly, if RSCU > 1.6 for a particular codon then it is considered as overrepresented and for a codon with RSCU < 0.6 it is considered as underrepresented (Sharp et al. 1988). RSCU value was used to identify the preferred codons. For each amino acid, the codon with the highest RSCU value is referred to as the preferred codon. Codons with RSCU value > 1 are designated as high-frequency codons while those with RSCU value < 1 are called low-frequency codons (dos Reis et al. 2003). (Supplementary file S3).

### Codon adaptability index (CAI) and codon bias index (CBI)

Codon adaptability index **(**CAI), a numerical measure for estimating the usage frequency of preferred codons among highly expressed genes was first developed by Sharp and Li 1987. CAI values range from 0–1; the elevated CAI values indicate higher adaptation, expressivity, and codon usage bias and vice versa (Sharp and Li 1986). Higher CAI values are shown by highly expressed genes like ribosomal proteins, transcription and translation factors, etc. CAI value is widely used in biological research to estimate cellular protein levels and translational accuracy (Vasanthi and Dass 2018). CBI is a quantitative measure that determines the extent to which preferred codons are used in a gene. It determines the level to which a gene uses highly expressed codons (Choudhury et al. 2017).

### Hydrophobicity (GRAVY) and Aromaticity (AROMO) analysis

CUB pattern of an organism may be influenced by the properties of proteins such as Hydrophobicity (GRAVY) and Aromaticity (AROMO). GRAVY value determines the nature of protein i.e. hydrophilic or hydrophobic. Positive GRAVY value indicates hydrophobic amino acid, while negative GRAVY score indicates the presence of hydrophilic amino acids (Kyte and Doolittle 1982). AROMO values of a protein determine the frequency of aromatic amino acids like phenylalanine, tyrosine, and tryptophan (Chen et al. 2014a, b). GRAVY and AROMO values of amino acids were used to determine the effect of natural selection on CUB by deriving a correlation between GRAVY, AROMO, and GC%, GC3%, Nc, CAI, etc.

### Effective number of codons (ENC)

Effective Number of Codons (ENC), an index used to measure the bias in usage of synonymous codons was first proposed by Wright (1990). Its values range from 20 to 61; ENC = 20 represents absolute bias i.e. only one of the available synonymous codons encode a given amino acid, while ENC = 61 indicates no bias i.e. all the synonymous codons code equally for a particular amino acid. Moreover, ENC < 35 indicates significant CUB in genes and genomes (Mensah et al., 2019).

### ENC plot (ENC vs GC3)

ENC values (as ordinate) were plotted against GC3 values (as abscissa) to obtain the expected slope of ENC curve called ENC plot (Supplementary file S4). ENC plot determines whether codon bias is only influenced by mutation pressure or by natural selection and other factors, and the relationship between CUB and base composition. For each CDS, if the ENC value falls on, near, or above the slope of the expected ENC line, mutational bias is considered as prime factor. Whereas, for values below the expected line, selection pressure and other factors play an important role (Novembre et al. 2002).

#### Parity (PR2) plot

The details of codon usage patterns among four-fold degenerate synonymous codons were determined in the host as well as pathogens using a PR2 plot. PR2 is a perpetuation of base pair rule (BPR) which assumes that A = T and G = C (where A + T + G + C = 1), provided no divergence exists between mutation and selection pressure of two DNA chains (Sueoka 1995). In particular, the PR2 bias assessed using just the third codon position is very informative (Sueoka 1999). In PR-2 plot, AT bias [A3/A3 + T3] and GC bias [G3/G3 + C3] were plotted as ordinate and abscissa, respectively (Supplementary file S5). The center of parity plot, where x = 0.5and y = 0.5 represent no deviation from BPR and a vector from the center represents the extent and direction of PR2 bias. Thus, in the PR2 analysis center point (0.5,0.5) of a plot where A = T, C = G, signifies that mutation pressure is the sole determinant of CUB. However, any deviation from the center indicates the influence of selection pressure and other factors in addition to mutation (Sueoka 2001).

#### Neutrality plot

The dominant factor (mutation pressure or natural selection) affecting codon usage bias in the host as well as pathogens was determined by analyzing the correlation between GC12 and GC3 through a scatterplot (Jia et al. 2015) (Supplementary file S6). In the neutrality plot, a statistically significant correlation between GC12 and GC3, a wide range of GC3 and regression slope close to 1 indicates that mutation pressure is largely influencing CUB (Liu et al. 2020). However, if the value of regression slope approaches 0, the effect of mutation decreases, whereas natural selection along with other minor factors play dominant role in influencing the codon usage bias.

#### Correspondence plot (CoA)

Correspondence plot (CoA) is a multivariate statistical analysis widely used to obtain a graphical representation of major trends in codon usage variation. In COA, all genes were distributed into 59- dimensions corresponding to the RSCU value of each codon except ATG, TGG, and three stop codons (Jia et al 2015).

#### Statistical analysis

Nucleotide composition and various other CUB indices like CAI, CBI, Fop, GRAVY, AROMO, etc. were calculated using CodonW (version 1.4.2) (http://codonw.sourceforge.net) and online computational biology tool (http://agnigarh.tezu.ernet.in/~ssankar/cbb_tu.html). INCA2.1 was used to calculate amino acid frequencies and codon frequencies. Correspondence analysis was performed using the software Past (Version 4.03) (Deb et al., 2020). Correlation analysis was performed using Minitab software (version 19.0). While, Anaconda 2 (Moura et al. 2005) was used for performing Codon context analysis.

## Results

### Identification of nematode and fungal pathogen

Among the nematode community the genera *M. incognita*, *Pratylenchus, Acrobeloides* and *Cephalenchus* showed a significant higher abundance in all the three rhizosphere soil samples of *W. somnifera.* While *Tylenchorhynchus* spp, *Helicotylenchus* spp and *Paratylenchus* spp were present less abundantly in all the three samples. Among 10 (ten) different identified genera of nematodes (*Criconemoids sp., Hemicriconemoides sp., Tylenchus sp., Ditylenchus sp., Tylenchorhynchus sp., Hoplolaimus sp., Helicotylenchus sp., Pratylenchus sp., Meloidogyne sp., Radinaphelenchus sp*.) root-knot nematode *M. incognita* was found to be the most dominant in our study.

The isolation frequency of RS-1 fungal strain was found to be maximum among all the fungal isolates of *W. somnifera.* Further, RS-1 was identified based on morphological and molecular characteristics. Macroscopically it was observed that the isolate initially formed grey coloured colonies that turned green after sporulation. Reverse of the colony was black in color. Microscopically, the isolate formed conidia which were obpyriform in shape with pointed beak. Both longitudinal and vertical septa were present in the conidia. These morphological features were compared to the literature and the isolate was identified as species of *Alternaria*. Further, sequence analysis of ITS1-5.8-ITS2 ribosomal DNA region was used to identify fungal isolate up to species level. BLAST results displayed 99.9% similarity with *A. alternata*. The sequence was submitted to Genbank under accession number MW741555.

### Nucleotide composition analysis

Comparative analysis of nucleotide composition was carried out in order to study its influence on CUB operative in host plants and its associated pathogens. We observed AT-biasness in the genome of *W. somnifera* and its nematode pathogen *M. incognita* as the mean AT% was found to be high. This further indicates A and T ending codons were more frequently used in coding sequences of these organisms. While fungal pathogen *A. alternata* showed higher mean GC% revealing that its coding sequences preferably contained more G and C ending codons, thus demonstrating a GC-biased genome (Table 1). Investigation of A, T, G and C content revealed that T% was highest in the host plant and its nematode pathogen suggesting an overall bias towards T ending codons in coding sequences of these organisms. In fungal pathogen, overall C% was highest indicating the prevalence of C- ending codons in selected coding sequences. Analysis of GC% content at three codon positions (GC1, GC2, GC3) revealed that GC1% was highest in the host plant and nematode as compared to GC2 and GC3 percentage. While in the fungal pathogen the percentage of GC at third position was maximum in comparison to GC percentage at first and second codon position.

**Table 1:** Nucleotide composition analysis for the coding sequences of the host plant *Withania somnifera* and the pathogens *Meloidogyne incognita* and *Alternaria alternata*

Nucleotide composition	*Withania somnifera*	*Meloidogyne incognita*	*Alternaria alternata*
GC%	40.31470588	37.8775625	54.1652657
AT%	59.68529412	62.1224375	45.8347343
GC1%	48.52214286	47.29825	56.22657005
GC2%	39.45558824	38.5460625	44.08241546
GC3%	32.96693277	27.787875	62.2126087
T3%	44.72726891	50.5935	26.51966184
C3%	19.66911765	17.2458125	45.57270531
A3%	38.11516807	44.3554375	21.49613527
G3%	20.61340336	16.7600625	29.9726087
AT3%	67.03306723	72.212125	37.7873913
AT2%	60.54441176	61.4539375	55.91758454
CAI	0.186306723	0.21084375	0.255690821
CBI	− 0.06837395	− 0.0964625	0.114608696
Fop	0.367945378	0.37203125	0.487347826
Nc	49.04970085	44.266125	50.60208738
L-sym	310.1008403	281.59375	589.4347826
L-aa	323.697479	291.475	612.7004831
Gravy	0.114367441	− 0.325850569	− 0.309608696
Aromo	0.114272277	0.096412469	0.081127952

### Codon usage bias analysis

In the present study the average ENC values for *W. somnifera*, *M. incognita* and *A. alternata* were found to be 40.74%, 38.38% and 46.59% respectively. High ENC values in all the three organisms indicate weak codon bias and neither of them have preferential codon usage.

CAI is a hallmark of the expressivity of genes. CAI output revealed that mean CAI values were 0.186, 0.210 and 0.255 for *W. somnifera, M. incognita* and *A. alternata* respectively. The high CAI value of fungal pathogen points towards its better adaptability to the host plant than the nematode pathogen (Nambou and Anakpa 2020). The extent of the usage of highly represented codons was determined using CBI analysis.

CBI values of less than zero in *W. somnifera* (-0.068) and *M. incognita* (-0.096) indicated random usage of non-preferred codons. While a CBI value close to zero in *A. alternata* (0.114) indicates random usage of both preferred and non-preferred codons.

Comparison of hydrophobicity (GRAVY) and aromaticity (AROMO) values of pathogens and host plant revealed that both pathogens (*M. incognita* and *A. alternata*) showed approximately equal proportion of hydrophilic amino acids as indicated by GRAVY values (-0.325 and -0.309) and also a similar amount of aromatic amino acids as shown by AROMO values (0.096 and 0.081) respectively. While host plant showed slightly lower proportion hydrophilic amino acids and slightly higher proportion of aromatic amino acids indicated by GRAVY (0.114) and AROMO (0.114) values (Table 2).

**Table 2:** Various codon usage bias indices values of *Withania somnifera* and the pathogens *Meloidogyne incognita* and *Alternaria alternata*

Species	ENC	CBI	CAI	GRAVY	AROMO	Fop
*Withania somnifera*	40.7434	− 0.0683	0.1863	0.1143	0.1142	0.3679
*Meloidogyne incognita*	38.3821	− 0.0964	0.2108	− 0.3258	0.0964	0.3720
*Alternaria alternata*	46.5971	0.1146	0.2556	− 0.3096	0.0811	0.4873

### RSCU and preferred codon analysis

RSCU value analysis revealed the preference of certain codons over others in all the selected organisms (Table 3). On comparing the preferred codon usage of host and pathogens, we observed that host plant *W. somnifera* and nematode pathogen *M. incognita* showed similarity in preferred codon usage for 17 amino acids except for amino acid Pro. In *W. somnifera* the preferred codon for amino acid Pro was CCU while *M. incognita* preferred to use CCA. It was also observed that most of the preferred codons used by host plant and nematode were A/U ending, predominately U-ending. Conversely, considerable deviation was observed between fungal pathogen and host plant over the preferred codon usage as both showed no similarity in the preferred codon usage with respect to any codon.

**Table 3:** Relative synonymous codon usage bias analysis for the coding sequences of *Withania somnifera* and the pathogens *Meloidogyne incognita* and *Alternaria alternata* (All the preferred codons were highlighted in bold)

Amino Acid	Synonymous codon	*Withania somnifera*	*Meloidogyne incognita*	*Alternaria alternata*
Ala	GCU	**1.759**	**1.762**	1.106
	GCC	0.698	0.575	**1.353**
	GCA	1.212	1.317	0.837
	GCG	0.331	0.296	0.704
Cys	UGU	**1.257**	**1.151**	0.560
	UGC	0.608	0.587	**1.305**
Asp	GAU	**1.508**	**1.425**	0.763
	GAC	0.492	0.525	**1.227**
Glu	GAA	**1.389**	**1.393**	0.670
	GAG	0.611	0.520	**1.330**
Phe	UUU	**1.334**	**1.377**	0.505
	UUC	0.666	0.623	**1.495**
Gly	GGU	1.251	1.023	1.088
	GGC	0.511	0.688	**1.556**
	GGA	**1.538**	**1.838**	1.003
	GGG	0.700	0.451	0.354
His	CAU	**1.393**	**1.356**	0.355
	CAC	0.532	0.506	**0.641**
Ile	AUU	**1.516**	**1.918**	0.937
	AUC	0.635	0.470	**1.679**
	AUA	0.849	0.612	0.384
Lys	AAA	**1.337**	**0.671**	0.475
	AAG	0.638	0.329	**1.525**
Leu	UUA	**1.705**	**1.957**	0.258
	UUG	1.252	1.444	0.783
	CUU	1.412	1.504	1.217
	CUC	0.480	0.465	**1.932**
	CUA	0.687	0.424	0.503
	CUG	0.464	0.205	1.307
Met	AUG	1.000	0.988	1.000
Asn	AAU	**1.405**	**1.440**	0.518
	AAC	0.569	0.498	**1.482**
Pro	CCU	**1.433**	1.407	1.026
	CCC	0.713	0.443	**1.255**
	CCA	1.278	**1.825**	0.967
	CCG	0.493	0.325	0.753
Gln	CAA	**1.394**	**1.574**	0.741
	CAG	0.606	0.389	**1.250**
Arg	CGU	1.438	1.500	1.121
	CGC	0.463	0.567	**1.998**
	CGA	1.229	1.341	0.966
	CGG	0.406	0.228	0.554
	AGA	**1.701**	**1.791**	0.600
	AGG	0.762	0.385	0.761
Ser	UCU	**1.718**	**1.823**	1.039
	UCC	0.949	0.572	1.225
	UCA	1.255	1.483	0.867
	UCG	0.441	0.438	1.045
	AGU	1.139	1.071	0.571
	AGC	0.499	0.613	**1.253**
Thr	ACU	**1.559**	**1.598**	0.905
	ACC	0.806	0.547	**1.521**
	ACA	1.239	1.501	0.901
	ACG	0.397	0.304	0.673
Val	GUU	**1.545**	**2.216**	1.012
	GUG	0.486	0.521	**1.752**
	GUA	1.283	0.717	0.572
	GUC	0.685	0.545	0.664
Trp	UGG	0.929	0.725	0.981
**Ter**	**UAA**	**1.576**	**1.988**	**1.232**
	UAG	0.731	0.394	1.130
	UGA	0.693	0.619	0.638
Tyr	UAU	**1.458**	**1.479**	0.526
	UAC	0.491	0.459	**1.474**

Uniqueness in the usage of preferred codons was observed in all the three organisms only with respect to amino acid Pro. *W. somnifera* preferred CCU codon for Pro, *M. incognita* favored CCA while *A. alternata* had chosen CCC codon for Pro.

In addition, we also observed that *W. somnifera* and *M. incognita* showed similarity related to overrepresented codons for amino acids Ala (GCU), Leu (UUA), Arg (AGA), Ser (UCU) and underrepresented codons for amino acids Ala (GCG), Asp (GAC), His (CAC), Leu (CUC, CUG), Asn (AAC), Pro (CCG), Arg (CGC), Ser (UCG) Thr (ACG), Val (GUG) and Tyr (UAC). While such similarity was observed in *W. somnifera* and *A. alternata* only with respect to underrepresented codon for one amino acid i.e. Arg (CGG). Further we found that overrepresented codons common in *W. somnifera* and *M. incognita* were mostly A/U-ending whereas underrepresented codons were C-ending.

### ENC plot analysis

**Fig. 1:**
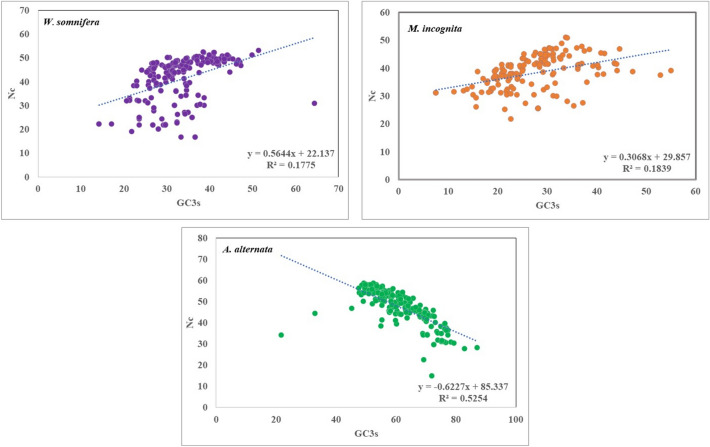
ENC-GC3 plot depicting the relationship between the effective number of codons (ENC) values and GC content at the third synonymous codon position (GC3) for host plant *W. somnifera* (**A**) and pathogens *M. incognita* (**B**) and *A. alternata* (**C**)

ENC/GC3 plot was generated to depict the influence of mutation pressure and natural selection on codon usage bias. ENC/GC3 plot analysis of all the three organisms (*W. somnifera*, *M. incognita and A. alternata*) revealed similarity in positioning of genes with respect to expected ENC line. Overall, It was observed that most of the genes were concentrated above the expected ENC line indicating the dominant effect of mutation pressure on CUB while a few genes were scattered below the line that point towards the minor role of natural selection in addition to mutation pressure on CUB (Fig. 1).

### Parity plot analysis

**Fig. 2:**
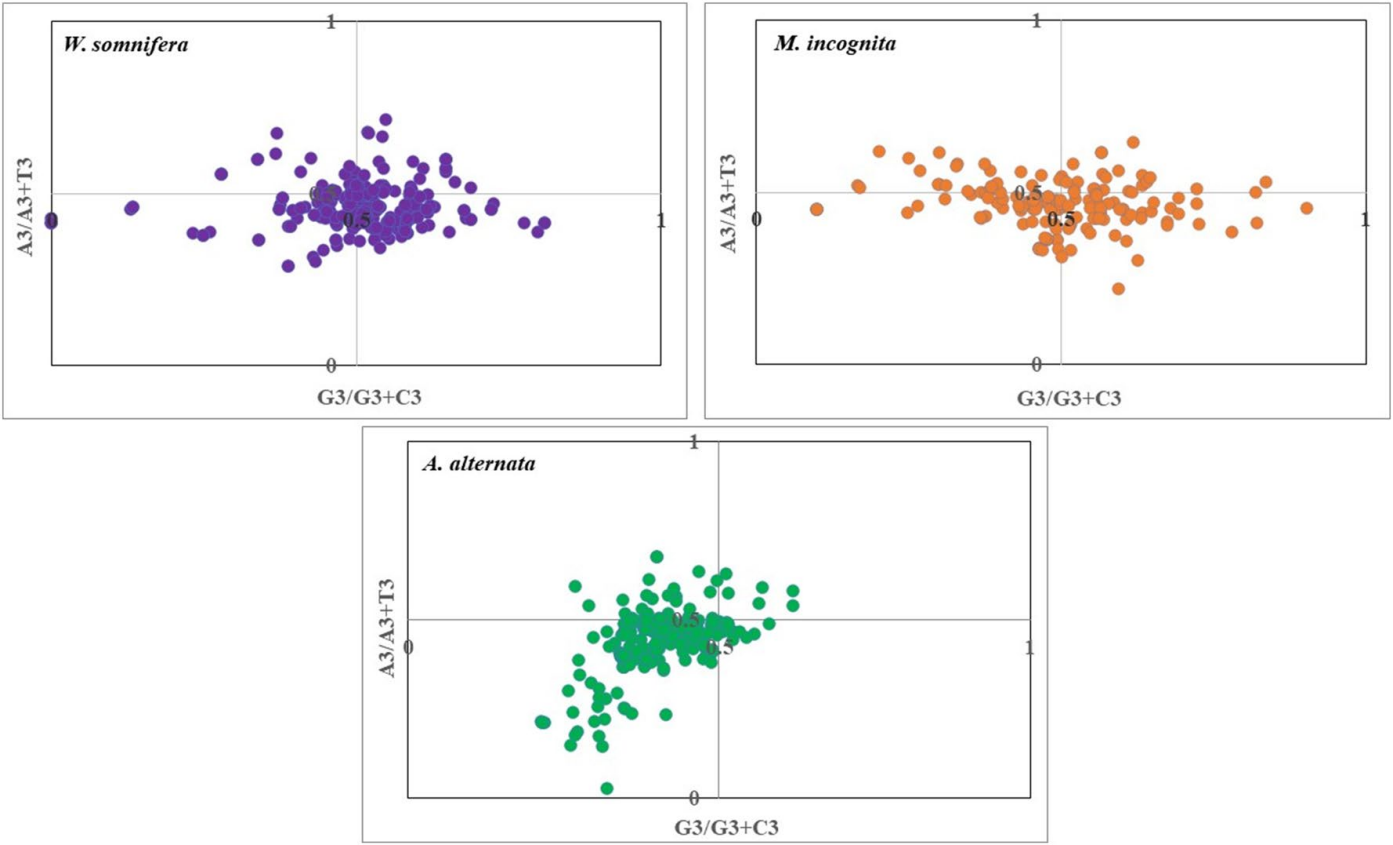
Parity Rule 2 (PR2)-bias plot between [A 3 / (A 3 + T 3) and G 3 / (G 3 + C 3)] of *W. somnifera* (**A**) and pathogens *M. incognita* (**B**) and *A. alternata* (**C**)

PR-2 bias plot analysis was used to examine the influence of mutational force and natural selection on the CUB of genes in the respective genomes. Parity plot analysis revealed the variation in the distribution pattern of genes in host and selected pathogens. We observed that for the host plant most data points were located in the lower right quadrant of the parity plot, indicating that T and G were the most frequently used nucleotides in the CDS (Fig. 2). Considering the general AT-bias in host along with parity plot results, there may exist a slight bias towards T-ending codons in selected coding sequences of the host. However, for pathogens, most of the genes were located in the lower-left quadrant of the PR-2 plot showing that bases T and C were most frequently used in their CDS. Given the general GC bias in *A. alternata* and general AT bias in *M. incognita*, there seems a bias towards C-ending and T-ending codons, respectively in their coding sequences. Overall, we observed an unequal usage of bases in all the three organisms suggesting the influence of both evolutionary forces mutational bias and natural selection on their CUB pattern (Chen et al. 2014a, b).

### Neutrality plot analysis

**Fig. 3:**
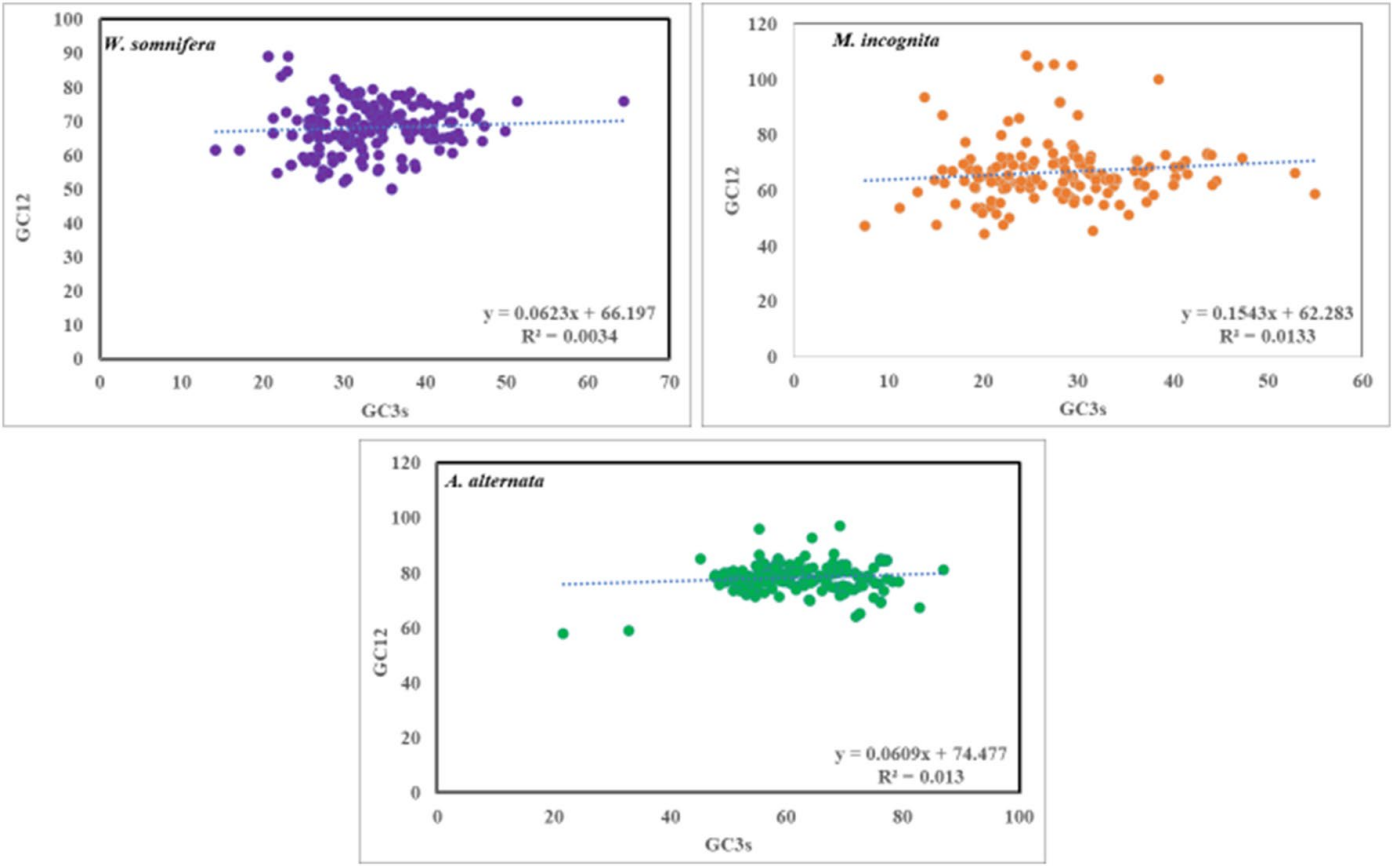
GC3s was plotted against GC12s. Neutrality plot for the coding sequences of the *W. somnifera* (**A**) and pathogens *M. incognita* (**B**) and *A. alternata* (**C**)

To accurately estimate the magnitude of mutational pressure and natural selection in shaping CUB in the host plant and its pathogens, the relationship between GC12% and GC3% was determined using a neutrality plot. The slopes of regression lines for *W. somnifera, M. incognita,* and *A. alternata* were found to be 0.06, 0.15, and 0.06 respectively. This suggests that the effect of relative neutrality (mutation pressure) on CUB was only 6%, 15%, and 6% in *W. somnifera, M. incognita* and *A. alternata* sequentially. While selection along with other compositional constraints predominately influence CUB in *W. somnifera* (94%), *M. incognita* (85%) and *A. alternata* (94%). Moreover, narrow range of GC3 distribution in all the three selected organisms further reinforced the role of natural selection on CUB operative in all the three organisms (Fig. 3). Correlation analysis revealed very low and statistically insignificant correlation between GC12 and GC3 in all the three selected organisms (Table 4).

**Table 4:** Correlation analysis between GC12 and GC3 values of the plant *Withania somnifera* and the pathogens *Meloidogyne incognita* and *Alternaria alternata*

GC12	GC3
*Withania somnifera*	r = 0.058, p = 0.372
*Meloidogyne incognita*	r = 0.115, p = 0.147
*Alternaria alternata*	r = 0.114, p = 0.102

### Correspondence analysis (COA)

**Fig. 4:**
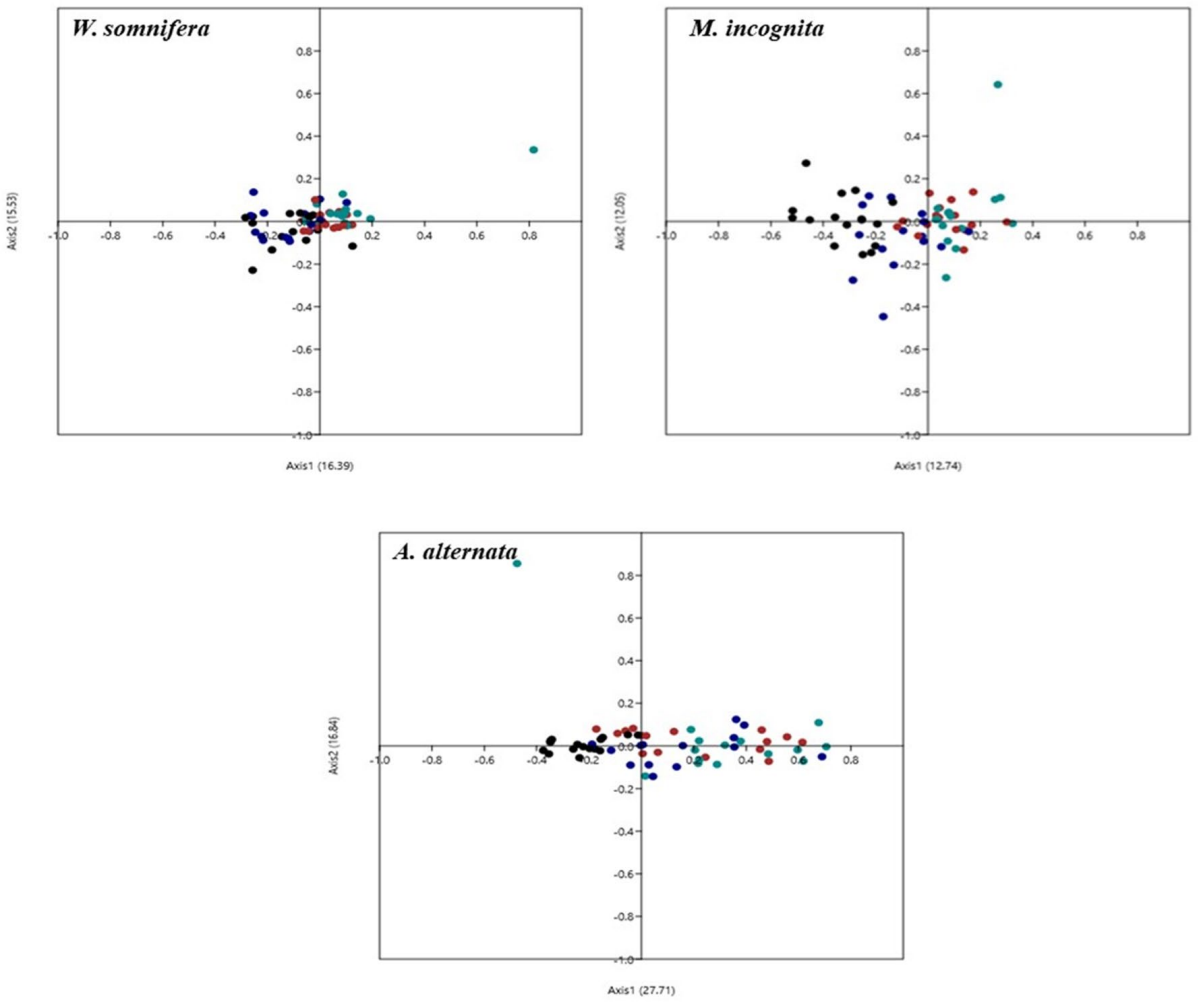
Correspondence analysis based on RSCU score of the coding sequences of *W. somnifera* (**A**) and pathogens *M. incognita* (**B**) and *A. alternata* (**C**). Black coloured data points represent GC– ending codons and brown coloured data points represent AT– ending codons in all the selected organisms

The variation in synonymous codon bias in the host as well as pathogens was assessed using correspondence analysis. COA analysis based on RSCU values of the host as well as the pathogens revealed that axis 1 and axis 2 were the major contributing axes responsible for the observed variance in *W. somnifera* (Axis 1: 16.39% and Axis 2: 15.53%), *M. incognita* (Axis 1: 12.74% Axis 2: 12.05%) and *A. alternata* (Axis 1: 27.71% and Axis 2: 16.84%) (Fig. 4). In *W. somnifera* both A/T (indicated in green and brown dots respectively) and G/C ending codons (indicated in blue and black dots respectively) were found to be concentrated towards the center of the plot. Whereas, in nematode pathogen most of the G/C ending codons were positioned around the center in the positive quadrant of the plot while few G/C ending codons were scattered away from central line in the negative quadrant. On the contrary, majority of A/T ending codons were located on the negative side of the plot and only few of them were distributed on the positive side of plot. This concentrated distribution of codons around the center of the plot indicated that mutation bias is mainly influencing the codon usage bias in host plant and nematode pathogen.

Conversely, in *A. alternata* majority of G/C ending codons were found near the central axis on negative side whereas most of the A/T ending codons were scattered along the positive quadrant of the plot in close proximity to the axis. This discrete distribution of codons in fungal pathogen indicates the influence of natural selection in addition to mutation on CUB. Overall, COA analysis reveals that mutation pressure is the main factor influencing CUB in host plant and nematode pathogen while scattered distribution of A/T and G/C ending codons along the axes in fungal pathogen indicates that in addition to mutation pressure, natural selection also significantly affects the CUB.

### Correlation analysis of CUB

The relationship between nucleotide composition and CUB indices (Nc/CAI used in the present study) were analyzed. In the host plant significant positive correlation of Nc with GC1, GC3, GC12, CAI and Fop was observed, but Nc had a significant negative correlation with AT3 (Table 5). Also, in the nematode pathogen Nc showed significant positive correlation with GC3 but a significant negative correlation was observed between AT3, T3, A3 and Nc. While in fungal pathogen Nc was significantly positively correlated with AT3, T3, A3 and significantly negatively correlated with GC3, CAI, and Fop. Interestingly, in both host plant and nematode pathogen Nc had a significant positive correlation with GC3 and significant negative correlation with AT3 indicating similarity in their correlation pattern. However, no such similarity in correlation of Nc with various nucleotide composition parameters was perceived in host plant and fungal pathogen.

**Table 5:** Correlation analysis between ENC and several other CUB indices of the plant *Withania somnifera* and the pathogens *Meloidogyne incognita* and *Alternaria alternata*

Nc	GC1	GC2	GC3	GC12	AT3	T3	A3	CAI	Fop
*Withania somnifera*	r = 0.373, p = 0.000	r = 0.085, p = 0.190	r = 0.421, p = 0.000	r = 0.339, p = 0.000	r = − 0.421, p = 0.000	r = − 0.111, p = 0.087	r = − 0.172, p = 0.008	r = 0.257, p = 0.000	r = 0.240, p = 0.000
*Meloidogyne incognita*	r = 0.122, p = 0.124	r = − 0.146, p = 0.066	r = 0.429, p = 0.000	r = 0.023, p = 0.777	r = − 0.429, p = 0.000	r = − 0.269, p = 0.001	r = − 0.258, p = 0.001	r = 0.027, p = 0.730	r = 0.041, p = 0.606
*Alternaria alternata*	r = − 0.022, p = 0.754	r = 0.076, p = 0.274	r =− 0.725, p = 0.000	r = 0.055, p = 0.434	r = 0.725 p = 0.000	r = 0.448, p = 0.000	r = 0.727, p = 0.000	r = − 0.743, p = 0.000	r = − 0.710, p = 0.000

In addition, correlation analysis of CAI with different CUB indices revealed that in *W. somnifera* CAI exhibits a significant positive correlation with GC1, GC3, T3, Nc, GC12 and Fop but it exhibits a significant negative correlation with AT3 and A3 (Table 6). In nematode pathogen, CAI showed significantly positive correlation with GC1, GC3, T3 and Fop but a significantly negative correlation with AT3 and A3. Whereas in *A. alternata*, CAI showed significant positive correlation with GC3 and Fop but it showed significant negative correlation with AT3, T3, A3 and Nc. Similarity in correlation pattern was observed in host plant and nematode pathogen as in both organisms CAI had a significant positive correlation with GC1, GC3, T3 and Fop but significant negative correlation with AT3 and A3. Further, host plant and fungal pathogen also shared similar correlation pattern as both showed significant positive correlation of CAI with GC3 and Fop while significant negative correlation of CAI with AT3 and A3. However, it is evident from the above analysis that nematode pathogen displays greater similarity with correlation pattern of host plant as compared to fungal pathogen. The frequency of optimal codon (Fop) is an important measure used to estimate CUB. In the present study, we observed a significant positive correlation between CAI and CBI/FOP in all the three organisms indicating a greater influence of gene expression on CUB.

**Table 6:** Correlation analysis between CAI and several other CUB indices of the plant *Withania somnifera* and the pathogens *Meloidogyne incognita* and *Alternaria alternata*

CAI	GC1	GC2	GC3	AT3	T3	A3	Nc	GC12	Fop
*Withania somnifera*	r = 0.336, p = 0.000	r = − 0.105, p = 0.105	r = 0.406, r = 0.000	r = − 0.406, r = 0.000	r = 0.152, p = 0.019	r = − 0.485, p = 0.000	r = 0.257, p = 0.000	r = 0.236, p = 0.000	r = 0.752, p = 0.000
*Meloidogyne incognita*	r = 0.273, p = 0.000	r = − 0.146, p = 0.066	r = 0.458, p = 0.000	r = − 0.458, p = 0.000	r = 0.188, p = 0.017	r = − 0.491, p = 0.000	r = 0.027, p = 0.730	r = 0.133, p = 0.093	r = 0.863, p = 0.000
*Alternaria alternata*	r = 0.051, p = 0.463	r = − 0.042, p = 0.544	r = 0.632, p = 0.000	r = − 0.632, p = 0.000	r = − 0.239, p = 0.001	r = − 0.790, p = 0.000	r = − 0.743, p = 0.000	r = 0.021, p = 0.768	r = 0.884, p = 0.000

Moreover, we also performed correlation analysis of the GRAVY and AROMO values of amino acids with different CUB parameters. GRAVY values showed a significant positive and negative correlation with ENC and GC, GC3 respectively in host plant while CAI showed insignificant correlation with GRAVY. In *M. incognita* GRAVY values showed significant negative correlation with GC3, ENC and CAI while an insignificant correlation of GC with GRAVY was observed. Further, in *A. alternata* only GC3% showed a significant negative correlation with GRAVY while other CUB parameters were insignificantly correlated (Table 7). Correlation between AROMO and CUB indices in host plant revealed a significant positive correlation of AROMO values with GC3 and ENC, significant negative correlation with GC and insignificant correlation with CAI while in nematode pathogen AROMO showed a significant negative correlation with ENC, GC3 and GC and an insignificant correlation with CAI. Similarly, AROMO values of *A. alternata* showed a negative correlation with GC% and an insignificant correlation with rest of CUB parameters (Table 8). Inclusively, significant correlation of different CUB parameters like ENC, GC3, CAI with GRAVY and AROMO values especially in host plant and nematode pathogen suggests that CUB in these organisms is considerably influenced by hydrophobic and aromatic amino acids that further indicates the role of translational natural selection on CUB of these organisms (Chen et al. 2014a, b).

**Table 7:** Correlation analysis between GRAVY and several other CUB indices of the plant *Withania somnifera* and the pathogens *Meloidogyne incognita* and *Alternaria alternata*

GRAVY	GC%	GC3%	CAI	ENC
*Withania somnifera*	r = − 0.252, p = 0.000	r = − 0.220, p = 0.001	r = − 0.005, p = 0.935	r = 0.617, p = 0.000
*Meloidogyne incognita*	r = − 0.117, p = 0.141	r = − 0.177, p = 0.025	r = − 0.224, p = 0.004	r = − 0.337, p = 0.000
*Alternaria alternata*	r = − 0.075, p = 0.282	r = − 0.163, p = 0.019	r = − 0.062, p = 0.371	r = − 0.033, p = 0.641

**Table 8:** Correlation analysis between AROMO and several other CUB indices of the plant *Withania somnifera* and the pathogens *Meloidogyne incognita* and *Alternaria alternata*

AROMO	GC%	GC3%	CAI	ENC
*Withania somnifera*	r = − 0.246, p = 0.000	r = 0.137, p = 0.034	r = 0.005, p = 0.943	r = 0.254, p = 0.000
*Meloidogyne incognita*	r = − 0.264, p = 0.001	r = − 0.176, p = 0.026	r = − 0.114, p = 0.151	r = − 0.184, p = 0.020
*Alternaria alternata*	r = − 0.140, p = 0.044	r = 0.012, p = 0.862	r = 0.116, p = 0.096	r = − 0.038, p = 0.586

Overall, correlation analysis revealed greater similarity in CUB between host and nematode parasite as compared to the fungal parasite.

#### Codon context analysis

Variations in codon context in the top 20 high-frequency codon pairs out of the 64 × 64 codon pairs were analyzed in the selected host and pathogens. Our results revealed similarities in codon pairing trends in the host and both pathogens (Table 9). Majority of codon pairs in host as well as pathogens consisted of preferred codons (indicated in red colour). Moreover, codon pairs consisting of preferred codons were found to be more prevalent in *M. incognita* (20/20) followed by *W. somnifera* (18/20) and *A. alternata* (17/20). Although, most of top 20 high -frequency codon pairs consisted of non-identical codons. However, tendency towards the usage of identical synonymous codon pairs (highlighted with yellow colour) was also observed and it was found to be highest in pathogens i.e. *A. alternata* (5/20) and *M. incognita* (4/20) followed by host *W. somnifera* (3/20). We observed that the majority of the codon pairs in the host plant and the nematode pathogen consisted of A/U ending codons while in the fungal pathogen most of the codon pairs consisted of G/C ending codons. In addition, the present study also emphasized the absence of codons of certain amino acids among top 20 high -frequency codon pairs. We found that codons encoding amino acids His, Pro, Arg, Ser, Thr and Val were not present among the top 20 codon pairs in both the host and selected pathogens. Additionally, the host plant also showed the absence of codons encoding Cys amino acid while Gln and Tyr encoding codons were found to be absent in fungal pathogen. Whereas the codons for amino acids Ala, Cys, Gly and Tyr marked their absence in top 20 high -frequency codon pairs of nematode pathogen.

**Table 9:** Codon context analysis of top 20 codon pairs in *Withania somnifera* and the pathogens *Meloidogyne incognita* and *Alternaria alternata* (All the codon pairs comprised of preferred codons were highlighted in red colored font)

*Withania somnifera*	*Meloidogyne incognita*	*Alternaria alternata*
AAA-AAA	UUU-UUU	AAG-AAG
GAA-GAA	UUA-UUU	GAG-AAG
AUU-GAU	UUA-AUU	GAC-GAG
AAG-AAA	UUU-UUA	GUC-AAG
AUU-GAA	GAA-GAA	GAG-GAG
AUU-UUU	UAU-UUU	AUC-AAG
AAA-GAA	UUA-UUA	AAG-GAC
UUU-GAU	AUU-UUU	UUC-AAG
AUU-GCU	UUU-AUU	GAG-AUC
AAU-GAU	CAA-CAA	GGC-AAG
GAU-AUU	AAU-UUU	GAA-GAG
CUU-CUU	AAU-UUA	CUC-GAC
UAU-GAA	UUA-AAU	GAC-GAC
GAA-GAU	GAA-AUU	GAC-AAG
GAA-AAA	UUU-UAU	AAC-AAC
AAA-UAU	AUU-UUA	UUC-UUC
AAA-GAU	AAA-AAU	UUC-GAC
CAA-GAA	UUU-GAU	GCC-AUG
UUA-UUU	AAA-UAU	GAC-AUC
GGA-AAA	GAU-GAA	CUC-AAG

#### Amino acid composition analysis

**Fig. 5:**
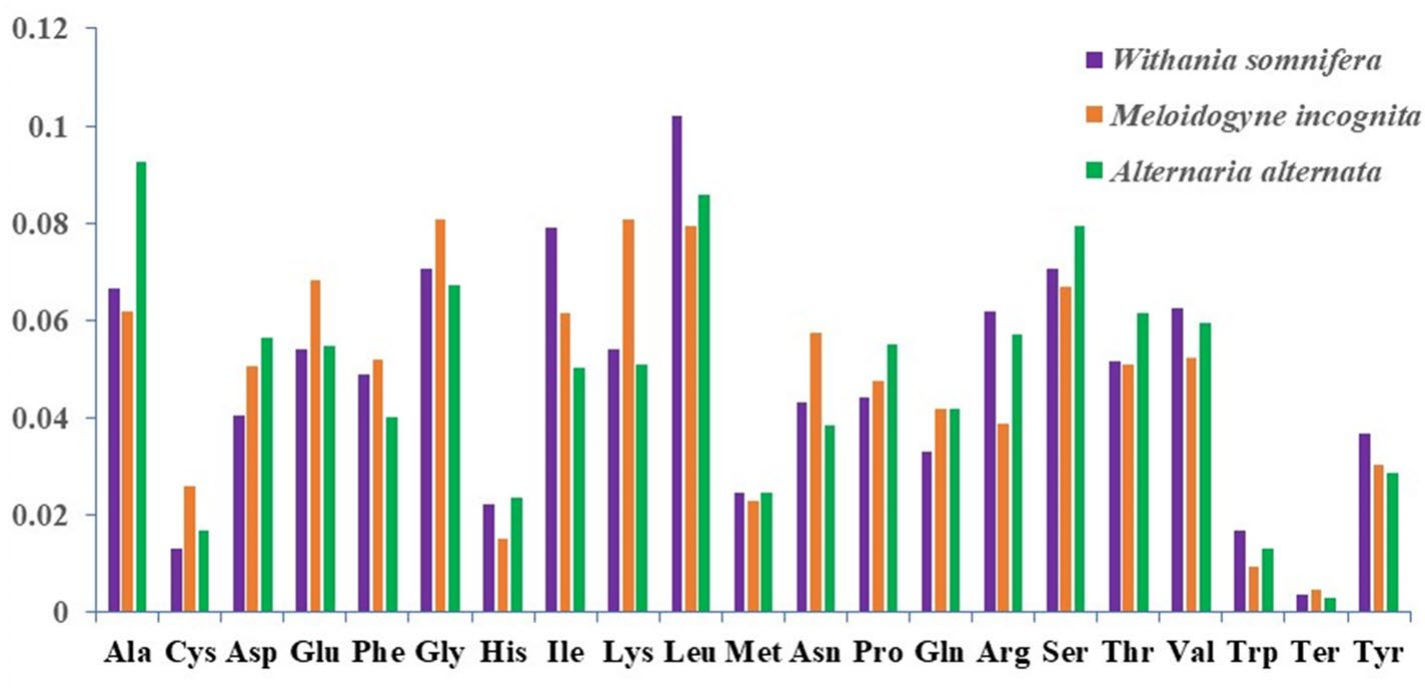
Comparison of amino acid usage between the pathogens (*M. incognita* and *A. alternata*) and host *W. somnifera*

Amino acid composition analysis revealed that in *W. somnifera* Leu was found to have maximum usage frequency followed by Ile, Gly and Ser. In *M. incognita,* amino acid Gly showed maximum frequency followed by Lys, Leu and Glu. Whereas, in *A. alternata* Ala showed the highest frequency followed by Leu, Ser and Gly. Detailed analysis further showed that in both the host as well as the pathogens, sixfold and fourfold amino acids were more frequent than threefold and twofold amino acids (Fig. 5). Furthermore, hydrophobic amino acids constituted a considerable proportion of complete amino acid content in both the plant and the pathogens.

## Discussion

Roots of the plant have imperatively been associated with numerous parasites. *M. incognita and* *A. alternata* are among the most common parasites found allied with the plant roots. *M. incognita,* a root-knot nematode has a worldwide distribution with numerous hosts (El-Sappah et al. 2019). It attacks the roots of plants deforming root cells and stimulating the formation of galls. Some of the crops that may be severely damaged by *M. incognita* includes tomato (Shukla et al. 2018), pepper (Carrillo-Fasio et al. 2021), grapevine (Wallis 2020), watermelon, cantaloupe, onion, pumpkin, cucumber (Mukhtar and Kayani 2019 etc. Root-knot nematodes also feed and multiply on many garden weeds. Similarly, *A. alternata* is one of the most prevalent phytopathogen that is responsible for causing different necrotic diseases in plants including black spot of strawberry (Fu et al. 2020) and Japanese pear, citrus brown spot on tangerine and grapefruit (Wu et al. 2020), leaf spot of lemon, brown spot of tobacco (Hatta et al. 2002; Jing et al. 2018) and leaf spot of *W. somnifera* (Singh et al. 2020). Among them, leaf spot disease in *W. somnifera* leads to huge loss in overall yield of plant. In the present study we found high abundance of *M. incognita* and *A. alternata* among various nematode and fungal isolates of *W. somnifera*. In view of their importance and dominant association with *W. somnifera,* the present study attempts to understand the intricate interaction between the medicinal plant *W. somnifera* and its selected pathogens (*M. incognita* and *A. alternata*) by comparative CUB analysis.

Nucleotide analysis revealed AT biasness in the genome of *W. somnifera* and its nematode pathogen, *M. incognita. W. somnifera* being a dicot plant showed similarity with the genomes of other dicot plants with respect to its observed AT biasness (Liu et al. 2020). Similarly, AT biasness in *M. incognita* has been found consistent with the previous reports (Mitreva et al. 2006) while GC biasness in genome of fungal pathogen *A. alternata* has also been confirmed with the earlier studies (Muthabathula et al. 2018; Roy and Staden 2019). The similarity in observed nucleotide composition between *W. somnifera* and *M. incognita* indicates that these species might have passed through a similar type of environmental stress in their ecological niches, emphasizing a possible evolutionary relationship between them (Mehmood et al. 2020; Gupta and Singh 2021). Relatedness in nucleotide composition between the host plant and nematode pathogen might be due to adaptive strategies of nematode for efficient translation under the host’s environment. However, we did not find any significant pattern of relationship between the host and fungal nucleotide composition. Therefore, other than CUB some other factors might have played a significant role in establishing the pathogenesis of *A. alternata* in *W. somnifera.*

The results of RSCU analysis with AT rich preferred codons in plant and *M. incognita* while dominance of GC ending codons in *A. alternata* supports the results of nucleotide composition. As previous reports indicate that in highly expressed genes, preference for C/G ending codons is associated with translational efficiency and fidelity (Dilucca et al. 2020). Therefore, GC richness and C ending codons in *A. alternata* might assist in high expression of pathogenicity related genes and increasing the severity of pathogenic interaction towards its host. In addition, other factors like virulence of the pathogen, host defense response, fungal population, and environmental conditions may be some other factors helping in the successful infestation of the host by fungal pathogen.

We found uniqueness in the use of preferred codons among host and selected pathogens with respect to amino acid Pro. It has been reported that in several pathogens amino acid Pro plays a critical role in pathogenesis by serving as an energy source, a critical respiratory substrate as well as a stress protectant (Christgen and Becker 2019). Consequently, overrepresentation of unique preferred codon encoding amino acid Pro in nematode pathogen might contribute to its better survival and adaptation under stressful conditions during different stages of pathogenesis. Additionally, disruption of proline metabolism in pathogens may be a useful approach to combat pathogenesis. Previous studies also report that accumulation of proline in plants is a common response to various abiotic and biotic stresses (Fabro et al. 2004). It further indicates that the unique preferred codon encoding Pro in host plant might contribute in pathogenic resistance by triggering proline accumulation.

The patterns of nucleotide composition and hence synonymous codon bias within and between genomes influence the amino acid usage (Cutter et al. 2006). However, amino acid composition and the evolution of proteins are partially influenced by the GC content of a genome whereas other factors like metabolic efficiency, molecular weight, and protein structure also play an important role (Du 2018). We observed Leu as the most abundant amino acid in *W. somnifera* as supported by previous studies in the chloroplast genome of *W. somnifera* (Mehmood et al. 2020). In plants LLR motifs, encoded by R genes are largely made of Leu that provide resistance to plants against bacterial, fungal and even nematode pathogens (Dangl and Jones 2001). In addition, we also found Ser to be the fourth most abundant amino acid in *W. somnifera*. It has been previously reported that high expression of serine protease inhibitors in underground parts of solanaceous plants help in the defense against Root-knot nematode (RNK) infection (Trudgill and Blok 2001). Therefore, the higher level of serine expression is an important adaptation of the host plant against *M. incognita* infection. Thus pathogens might possibly have devised some mechanism to counteract the resistance implicated by these amino acids in the plant in order to establish pathogenic relation with the host.

Gly was found to be the most abundant amino acid in *M. incognita.* As indicated by the S/C score, Gly (1.00) is biosynthetically less costly. Higher expression of Gly may reduce the metabolic burden on the nematode pathogen and may facilitate the higher expression of pathogenicity and vitality related genes. Similarly, the abundance of Ala (S/C score 4.76) in the fungal pathogen reinforced the cost minimization strategy of pathogens for increased expression of vitality and robustness related genes during pathogenic interaction.

In codon context analysis of all the three organisms, the absence of preferred codons encoding amino acids like Ala, Cys, Gln, Gly, His, Pro, Arg, Tyr, Trp, Thr was observed. The plausible reason for their absence might be attributed to higher biosynthetic cost incurred by these amino acids. In addition, the tendency towards the usage of homogenous preferred codon pairs especially in pathogens *A. alternata* (5/20) and *M. Incognita* (4/20 codon pairs) might be ascribed to cost minimization strategy of the pathogens. Previous studies report that homogenous codon pairs require much less energy as compared to heterogeneous codon pairs during protein synthesis (Deb et al. 2021b, a).

The ENC, parity (PR-2) and neutrality plot analysis revealed the dominant effect of natural selection while COA suggests mutational pressure to be the main factor affecting CUB in all three organisms. Overall, we can conclude that both the evolutionary forces *viz* natural selection and mutation influences the CUB operative in host plant and its associated pathogens. However, predominant role of natural selection over mutation is in consensus with the previous studies conducted on plants like *Oryza sativa* (0.143) and *Zea mays* (0.140) that have also reported natural selection as the main factor influencing CUB (Duret and Mouchiroud 1999; Liu et al. 2004).

## Conclusion

The present study highlight the intricacies of codon and amino acid usage in highly potent medicinal plant *W. somnifera* and two of its economically important pathogens viz. *M. incognita* (nematode) and *A. alternata* (fungus). Extensive comparative study of CUB in all three selected organisms revealed AT-biasness in the genome of  *W. somnifera* and *M. incognita* while GC-biasness in *A. alternata *. This indicates  more host-specific codon usage patterns and codon usage adaptability in *M. incognita* as compared to *A. alternata* towards their host*.* Similarity in their CUB further suggests that *M. incognita* might have coevolved with *W. somnifera* and CUB may possibly play an important role in successful plant–nematode interaction and pathogenesis. High ENC values (ENC > 35) revealed weaker codon usage bias in all the three selected organisms. Furthermore, the results of different indices and plots revealed the influence of both evolutionary forces on CUB of host and pathogens with predominance of natural selection. This study will be the first to unravel the role of CUB operative in *W. somnifera* and its integrated pathogens. Moreover, our study would lay a foundation for the future research on other pathogens associated with *W. somnifera*.

## Supplementary Information

Below is the link to the electronic supplementary material.

Supplementary file1 (XLSX 503 kb)

Supplementary file2 (XLSX 20 kb)

Supplementary file3 (XLSX 250 kb)

Supplementary file4 (XLSX 61 kb)

Supplementary file5 (XLSX 125 kb)

Supplementary file6 (XLSX 82 kb)
